# ESCRTs in plant abiotic stresses

**DOI:** 10.1042/EBC20253050

**Published:** 2026-03-16

**Authors:** Cuo Mei, Qi Xie, Feifei Yu

**Affiliations:** 1 State Key Laboratory of Maize Bio-breeding, College of Grassland Science and Technology, China Agricultural University, Beijing, 100193, China; 2 University of Chinese Academy of Sciences, Beijing, 100049, China; 3 State Key Laboratory of Crop Germplasm Innovation and Molecular Breeding, Syngenta Group China, Beijing, 102206, China; 4 Institute of Genetics and Developmental Biology, The Innovative Academy of Seed Design, Chinese Academy of Sciences, Beijing, 100101, China

**Keywords:** ABA, ESCRT, FREE1/FYVE1, ubiquitination, VPS23A

## Abstract

The endosomal sorting complex required for transport (ESCRT) is a conserved molecular machinery that plays fundamental roles in the cellular endomembrane network. Functioning as a core mechanism, ESCRTs recognize and sort ubiquitinated membrane proteins, which are subsequently sequestered into vacuoles (lysosomes in other eukaryotes) for degradation by luminal proteases or recycled from endomembranes to the plasma membrane for functional reuse. Through these processes, the ESCRT machinery acts as a critical regulator of plant development and stress adaptation. Recent studies on plant ESCRT components, particularly VPS23A and FREE1, have identified their key roles in abiotic stress responses, with a focus on their modulation of abscisic acid (ABA) signaling pathways. Additionally, post-translational modifications including ubiquitination and phosphorylation have been shown to play pivotal roles in these regulatory processes. Notably, FREE1 has been identified to mediate endosome membrane bending and scission independently of the ESCRT machinery, a mechanism crucial for plant responses to osmotic stress. This review summarizes and discusses recent advances in ESCRT-mediated signaling in plant abiotic stress responses, aiming to highlight the fundamental roles of ESCRTs in plant biology and provide key targets for molecular breeding of abiotic stress-tolerant crops.

## Introduction

**Figure 1:**
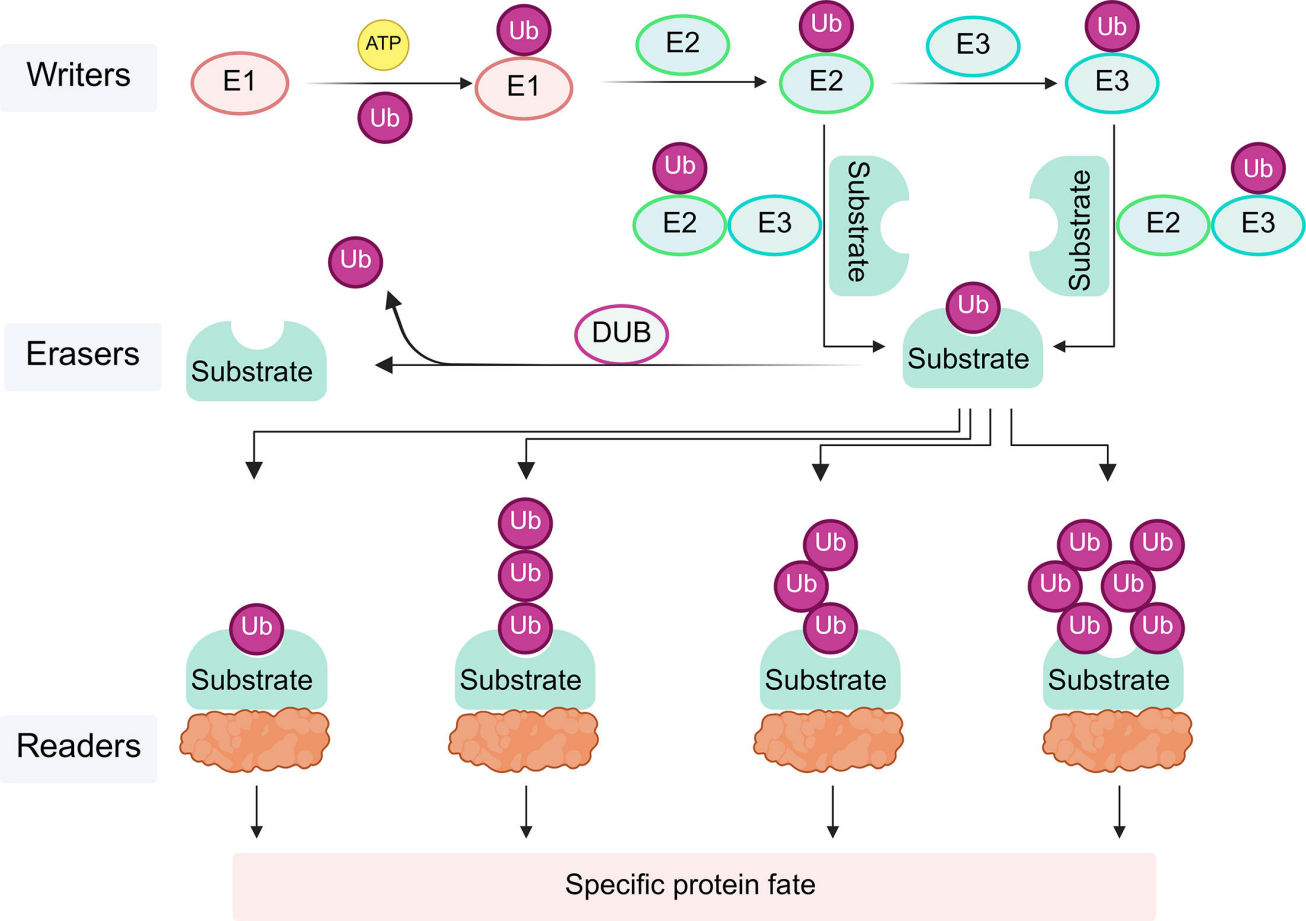
Ubiquitination modification regulates protein fate.

Membrane trafficking mediated by the endomembrane system is critical for cellular proteins to function at the correct location with proper abundance. The endosomal sorting complex required for transport (ESCRT) machinery represents an evolutionarily conserved multi-subunit membrane remodeling complex. It plays essential roles in multivesicular body (MVB) biogenesis and the sorting of membrane proteins into intraluminal vesicles (ILVs). MVBs are specialized organelles formed by the encapsulation of small vesicles within larger endosomal compartments, acting as a key intermediate in lysosome biogenesis and generating ILVs via inward membrane invagination. The ILVs are critical either for degradation upon MVB–vacuole/lysosome fusion or for subcellular re-localization. For example, tumor cells can enhance their resistance to cytotoxic T lymphocytes (CTLs) and natural killer cells via ESCRT-mediated membrane repair [1]. The E3 ubiquitin ligase lsterin interacts with α-syn (a major contributor to Parkinson’s disease) and facilitates its K27-linked ubiquitination, which promotes the protein’s sorting into the endosome pathway for subsequent degradation via an ESCRT-dependent mechanism [2]. It should be noted that ubiquitination modifications of cargo proteins are essential for their recognition and sorting by ESCRTs. The classic process of ubiquitination modification can be summarized into three categories: writers (most E3s, or rarely E2s and E3s), erasers (deubiquitinases, DUBs), and readers [3]. The ubiquitination process is mediated by a cascade enzymatic reaction involving E1, E2, and E3 (Figure 1) [4]. Conversely, DUBs catalyze the hydrolysis of the isopeptide bond between the Ub chain and proteinaceous substrate, enabling Ub recycling and regulating protein modifications [5].

Ubiquitination acts as a ‘writer’. Overview of the ubiquitination modification process: Protein ubiquitination modification is a three-step, ATP-dependent cascade reaction. Ubiquitin molecules are activated by E1 (ubiquitin-activating enzyme), and a high-energy thioester bond is formed to covalently link the C-terminal glycine of ubiquitin to the active-site cysteine residue of E1. The activated ubiquitin then is transferred from E1 to the active-site cysteine residue of E2 (ubiquitin-conjugating enzyme), forming a new high-energy thioester bond. Finally, under the action of E3 (ubiquitin ligase), ubiquitin is transferred from E2 to the target substrate protein, completing the modification. Uniquitination is reversible. Deubiquitinating enzymes (DUBs) function as ‘erasers’, removing ubiquitin from proteins either for recycling or to regulate protein modifications. ‘Readers’ identify ubiquitin-tagged proteins and determine their fate, such as degradation, relocation, or complex formation, depending on the type of ubiquitin chain attached.

Although modulation of the ESCRT machine has been reported to be involved in plant growth, development, and responses to abiotic and biotic stresses [6], the detailed mechanism remains poorly understood. Most studies of ESCRTs have focused on their roles in membrane biogenesis and trafficking processes, while the roles of ESCRT components and the system in plant functional biology remain poorly characterized. As global extreme climate events occur more frequently, environmental stresses (including drought, high salinity, and saline-alkaline soils) pose severe challenges to crop growth and yield [7–10]. Crop yield losses due to drought stress are equivalent in magnitude to those caused by all pathogens together [11]. Excitingly, the roles of ESCRTs in plant abiotic stress adaptation have attracted increasing attention over the past decade, with significant breakthroughs achieved. In this review, we summarize these studies and emphasize the roles of two key ESCRT-I components, VPS23A and FREE1 (also termed FYVE 1, FYVE domain protein required for endosomal sorting one), which are responsible for recognizing ubiquitinated cargo proteins, in plant abiotic stress tolerance.

## ESCRTs in plants

The plant ESCRT machinery includes ESCRT-I, -II, -III, and the VPS4/SKD1 (vacuolar protein sorting 4/suppressor of K ^+^ transport growth defect 1)-LIP5 (Lyst-interacting protein 5) subcomplexes. Unlike ESCRTs in mammals, no canonical ESCRT-0 subunit has been identified. However, the Arabidopsis genome encodes 9 TOM1-like proteins (TOLs) [12]. TOL proteins contain conserved VHS [VPS27, HRS (hepatocyte growth factor-regulated tyrosine kinase substrate)] and STAM (signal transducing adaptor molecule) domains, which are responsible for binding ubiquitinated cargo and performing functions similar to those of ESCRT-0 in mammals [12,13]. Arabidopsis contains three ESCRT-I subunits: VPS23 (homolog of tumor susceptibility gene-101 (TSG101) in mammals), VPS28, and VPS37, but lacks MVB12. Plant ESCRT-II is also composed of three subunits: VPS22, VPS25, and VPS26 [14]. Through the recognition and binding of VPS23 and VPS36, ubiquitinated proteins are transferred to ESCRT-I and ESCRT-II. ESCRT-III in Arabidopsis is highly conserved, including VPS2, VPS20, VPS24, SNF7, CHMP1, and ISTL1, as well as ALG-2 INTERACTING PROTEIN-X (ALIX), which interacts with SNF7 [13]. Through the interaction between VPS25 and VPS20, the ESCRT-III complex is activated and forms invaginations on MVB membranes. The ATP-dependent ESCRT machinery mediates the capture and engulfment of membrane-bound cargo proteins through invagination and scission of MVB membranes to form ILVs. The AAA ATPase VPS4/SKD1-LIP5 complexes hydrolyze ATP to dissociate the ESCRT-III complex from the endosomal membrane into the cytoplasm for recycling.

**Figure 2:**
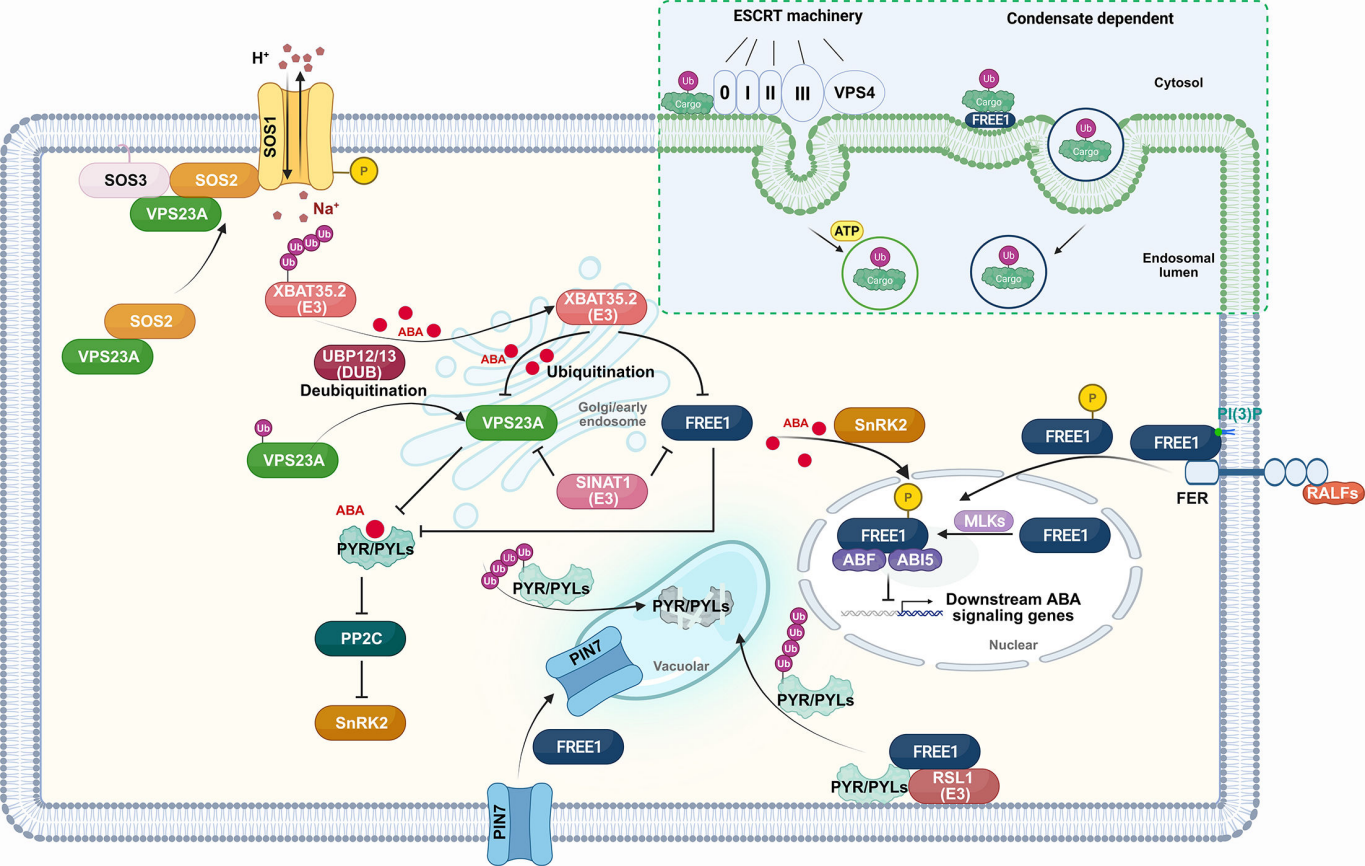
Function of VPS23A and FREE1 in plant abiotic stress response.

The plasma membrane and endomembranes of eukaryotic cells are highly dynamic mosaic-fluid structures that undergo continuous remodeling, including bulging, invagination, budding, fusion, and fission. ESCRT-mediated membrane dynamics processes are essential for virtually all major cellular functions, including nutrient uptake, signaling, pathogen defense, cell division, and migration in mammals [15,16]. Recently, liquid–liquid phase separation (LLPS) has been identified to function in endomembrane remodeling. Phase separation refers to the phenomenon within the cytoplasm whereby certain proteins and nucleic acids (such as DNA and RNA) spontaneously aggregate to form droplet-like structures, independent of membrane encapsulation. FREE1 exhibits strong phase separation ability due to its N-terminal intrinsically disordered region (IDR), a core structural feature that drives protein phase separation [17]. IDRs are defined as protein regions that lack stabled or ordered three-dimensional structures, making them more prone to form liquid-like states and induce phase separation while regulating its dynamics. Importantly, FREE1 forms liquid-like condensates that associate with membranes to drive ILVs formation. The line tension forces induced by condensate wetting, coupled with the membrane asymmetry conferred by FREE1, are sufficient to mediate scission of the membrane neck. While the ESCRT machinery is canonically required for membrane scission to complete ILV biogenesis, a process driven by membrane neck instability, FREE1 alone can mediate ILV formation via LLPS. This activity is independent of both the ESCRT machinery and ATP hydrolysis (Figure 2) [17].

The ubiquitination of ABA receptor PYL4 by RSL1 at the plasma membrane suggests that this receptor may be directed to the vacuolar degradation pathway. Components of the ESCRT complex, VPS23A and FREE1, interact with ABA receptor PYL4 to facilitate its degradation in vacuoles, thereby influencing receptor localization and protein stability. VPS23A and FREE1 are targeted by E3 ubiquitin ligase SINAT1 for ubiquitination and degradation. E3 ubiquitin ligase XBAT35.2 directly mediates K48-linked ubiquitination of VPS23A, promoting its degradation in response to ABA treatment and thereby relieving VPS23A-mediated inhibition of PYL4 protein activity. Deubiquitinases UBP12/13 protect VPS23A stability through deubiquitination and stabilize XBAT35.2 after ABA treatment, enabling fine-tuned regulation of the ABA signaling pathway in response to environmental stress. Additionally, VPS23A interacts with SOS2 and SOS3, enhancing their interaction. Under salt stress, VPS23A promotes the relocalization of SOS2 to the plasma membrane, activating antiporter SOS1 activity and improving salinity tolerance. FREE1 can be phosphorylated by SnRK2 and the RALF1 peptide-FERONIA complex, after which it translocates to the nucleus. FREE1 is also phosphorylated by MLKs in the nucleus; phosphorylated FREE1 interacts with ABF4 and ABI5, reducing their binding to cis-regulatory sequences of downstream ABA signaling pathway genes. Moreover, FREE1 promotes PIN7 localization in vacuoles and enhances plant tolerance to alkaline stress. The GREEN BOX indicates that, unlike the ESCRT machinery, FREE1 can form membrane-associated liquid-like condensates via liquid–liquid phase separation (LLPS), driving the formation of intraluminal vesicles. Condensate-wetting-induced membrane fission occurs without relying on traditional ESCRT machinery or ATP hydrolysis.

Furthermore, numerous plant-specific components involved in or associated with ESCRTs were identified (Table 1). Src homology-3 (SH3) domain-containing protein 2 (SH3P2) is an ESCRT-I and ubiquitin-binding protein that functions in intracellular trafficking; it may act similarly to ESCRT-0 in binding ubiquitinated cargo proteins [24]. Arabidopsis lacks the ESCRT-0 complex and ESCRT-I component MVB12, both present in mammals and yeast, yet absence does not disrupt the interaction network among core ESCRT components. Instead, several plant-specific ESCRT-associated proteins have been characterized, including the suppressor of FREE1, resurrection 1 (RST1) [29–32], FYVE4 [25], the positive regulator of SKD1 (PROS) [23], and the BRo1-domain protein as FREE1 suppressor (BRAF) [26]. ALIX/AtBRO1 (Arabidopsis apoptosis-linked gene-2 interacting protein X), an ESCRT-associated protein that interacts with CHMP4 subunits—facilitates cargo sorting and ILV formation. It also co-ordinates membrane transporter degradation and vacuole-related processes through interactions with retromer core components Vps26 and Vps29 [33]. Thus, while the plant ESCRT system is evolutionarily conserved, it has evolved distinct, plant-specific features.

**Table 1:** ESCRT components and their biological functions in plants

Regulators	Protein	Functional annotation	Refs
Functional analogies to ESCRT-0	TOM1-like proteins (TOLs)	Binding ubiquitinated cargo and performing functions similar to those of ESCRT-0 in mammals	[12]
ESCRT-I	VPS23	Endosomal sorting, vacuolar biogenesis, abiotic stress responses	[18]
	VPS28	Endosomal sorting	[19]
	VPS37	Endosomal sorting	[19]
	FREE1	MVB and vacuole biogenesis, endosomal sorting, abiotic stress responses	[12]
ESCRT-II	VPS22		[14]
	VPS25		[14]
	VPS26	MVB biogenesis, endosomal sorting	[14]
ESCRT-III and accessory proteins	VPS2	MVB biogenesis, endosomal sorting	[13]
	VPS20	MVB biogenesis, endosomal sorting	[13]
	VPS24	MVB biogenesis, endosomal sorting	[13]
	SNF7	MVB biogenesis, endosomal sorting	[13]
	CHMP1	MVB biogenesis, endosomal sorting	[13]
	ISTL1	MVB biogenesis	[13]
VPS4/SKD1- LIP5 subcomplexes	VPS4/SKD1	MVB biogenesis, endosomal sorting	[19]
	LIP5	MVB biogenesis, endosomal sorting	[20–22]
	PROS	Regulate SKD1 ATPase activity	[23]
Other ESCRT-related proteins	SH3P2	Intracellular trafficking	[24]
	FYVE4	MVB biogenesis, endosomal sorting	[25]
	BRAF	MVB biogenesis, membrane protein homeostasis	[26]
	ALIX/BRO1	Cargo sorting, ILV formation, membrane transporter degradation, and vacuole-related processes	[27,28]

## VPS23A in plant abiotic stress

Ubiquitinated membrane receptors are recognized and transported by the ESCRT machinery, then subsequently sequestered into vacuoles (lysosomes in other eukaryotes) for degradation by luminal proteases [19,34]. The ESCRT-I component VPS23A contains a ubiquitin-conjugating enzyme variant (UEV) domain that lacks the functional cysteine conserved in UBC domains but retains ubiquitin-binding activity and often modulates E2 function. Notably, VPS23A has been shown to recognize and sort soluble abscisic acid (ABA) receptors, which are ubiquitinated by the RING E3 ubiquitin ligase RING finger of seed longevity 1 (RSL1) at the plasma membrane, and thereby influencing plant responses to abiotic stress [18,35].

ABA plays a pivotal role in plant drought tolerance and stress adaptation, deserving it the designation of ‘stress phytohormone’ [36]. To investigate the role of Arabidopsis UBC E2 or UEV E2-like proteins in ABA signaling, phenotypic screening of mutants of these genes was conducted. Intriguingly, VPS23A was identified as a negative regulator of ABA signaling [7]. VPS23A interacts with PYR1/PYLs that carry K63-linked ubiquitination modifications. Further analysis confirmed that VPS23A modulates the subcellular localization and stability of PYR/PYL/RCAR-type ABA receptors through endosomal sorting to vacuole-mediated degradation [18]. This study advanced our understanding of how ESCRT components regulate the turnover of soluble receptors and their involvement in plant hormone signaling pathways.

In the course of characterizing VPS23A as a regulator of ABA signaling, VPS23A was found to be an unstable protein [18,37]. The UEV domain in VPS23A has been elucidated to recognize the tetrapeptide protein motif PS/TAP in both yeast and mammals [38,39]. To elucidate the factors modulating VPS23A, an E3 ubiquitin ligase XBAT35.2 that contains two PSAP motifs was uncovered. The interaction between VPS23A and XBAT35.2 results in the deposition of K48 polyubiquitin chains on VPS23A, accelerating its degradation via the 26S proteasomes. XBAT35.2 acts as a positive regulator of ABA signaling and drought stress responses by accelerating VPS23A turnover, thereby protecting ABA receptors [37]. This discovery further demonstrated crosstalk between the ubiquitin–proteasome system (UPS) and endosome–vacuole-mediated degradation pathways, which is crucial for modulating ABA signaling-mediated plant drought responses.

Ubiquitination of VPS23A is reversible. Interestingly, the deubiquitinases UBP12 and UBP13 were found to act synergistically with the E3 ubiquitin ligase XBAT35.2 to regulate VPS23A ubiquitination [5]. The *ubp12* and *ubp13* mutants display similar ABA sensitivity and drought tolerance phenotypes to *vps23a*. In addition to directly regulating VPS23A, UBP12 and UBP13 were found to deubiquitinate and stabilize XBAT35.2 in response to ABA [5]. Since UBP12/UBP13 play a protective role in ABA signaling by acting on both the negative factor VPS23A and the positive factor XBAT35.2, they might play a buffering role in the fine regulation of VPS23A ubiquitination and stability under normal and stressed conditions [5]. Thus, UBP12 and UBP13 are rheostatic regulators of ABA signaling, modulating it by directly and indirectly controlling the ubiquitination status and protein levels of the ESCRT component VPS23A. This provides not only new insights into the interplay among ESCRTs, UPS, and ABA pathways, but also insights into how ubiquitination and deubiquitination modifications finely regulate the transition between normal growth and stress responses.

In addition to its significant role in drought stress, VPS23A plays a notable part under salt stress responses. Historically, the classical Salt-Overly-Sensitive (SOS) signaling module has been well studied as a core mechanism for plant salt tolerance. This pathway primarily involves the plasma membrane (PM) Na^+^/H^+^ antiporter protein SOS1, along with calcium-signaling-related regulatory proteins SOS2 and SOS3 [40]. VPS23A was found to positively regulate plant salt tolerance by enhancing the activity of the SOS pathway [40]. Specifically, VPS23A interacts with both SOS2 and SOS3 but not with SOS1. Under salt stress, VPS23A positively regulates the redistribution of SOS2 to the plasma membrane, which then activates SOS1 antiporter activity, resulting in reduced Na^+^ accumulation in cells to protect plants from salt stress [40]. Arabidopsis seedlings exhibit stronger salt tolerance when expressing engineered membrane-bound SOS2 compared with SOS2 alone [40]. This finding highlights the importance of SOS2 being sorted to the plasma membrane to fulfill its function, offering a potential approach for engineering salt tolerance in crops. Although ABA plays key roles in plant salt stress responses, whether VPS23A-mediating sorting of SOS2 is connected to the ABA pathway remains an open question. Additionally, a recent report on SOS1 emphasizes its role in vacuolar sequestration of Na^+^ [41]; whether VPS23A functions in the vacuolar localization of SOS1 requires further investigation. Importantly, whether the function of VPS23A in drought and salt stress responses is dependent or independent of ESCRTs remains an open question.

## FREE1 in plant abiotic stress

Beyond VPS23A, another subunit deserving in-depth discussion is FREE1. As a plant-specific ESCRT-I component, FREE1 plays crucial roles not only in ABA signaling but also in auxin and target of rapamycin (TOR) signaling pathways, thereby contributing to plant tolerance to abiotic stresses. The ubiquitination of ABA receptors PYR1 and PYL4 by RSL1 at the plasma membrane suggests that these receptors may be directed to the vacuolar degradation pathway, with ubiquitination acting as an internalization signal for endocytic trafficking [35]. Subsequent studies revealed that, analogous to VPS23A, FREE1 interacts with RSL1-ABA receptor complexes to recruit ubiquitinated ABA receptors into the endosomal trafficking pathway [25]. Specifically, FREE1 recognizes ubiquitinated PYR1/PYL4 receptors and targets them for vacuolar degradation. Consistent with this mechanism, *FREE1* mutants accumulate PYR1/PYL4 proteins and display hypersensitivity to ABA treatment [23].

FREE1 function is further modulated by post-translational modifications, including ubiquitination (as with VPS23A) and phosphorylation—both of which are critical for a comprehensive understanding of FREE1-mediated stress regulatory networks. The E3 ubiquitin ligases SINATs have been shown to ubiquitinate both FREE1 and VPS23A, promoting their proteasomal degradation [42,43]. Additionally, SINAT-FREE1/VPS23A protein complexes can undergo co-degradation via the vacuolar pathway. Overexpression of SINATs accelerates FREE1 degradation, elevates PYL4 protein levels, and enhances ABA sensitivity [42,43]. Notably, during recovery from ABA treatment, SINAT proteins form homo- or hetero-oligomers that are predominantly degraded via the autophagic pathway in planta. This releases ESCRTs to remove PYR1 and PYL4, thereby terminating ABA signaling. Thus, SINATs play a key role in deactivating ABA signaling.

Several kinases have been identified to phosphorylate FREE1, including SNF1-related protein kinase 2 s (SnRK2s), the receptor-like kinase FERONIA (FER), MUT9-like kinases 1–4 (MLKs 1–4) and SOS2. FREE1 was first discovered to be phosphorylated by two key kinases in ABA core signaling: SnRK2.2 and SnRK2.3 (but not SnRK2.6) upon ABA treatment. Subsequently, phosphorylated FREE1 at residues Ser530 and Ser533 is shuttled to the nucleus, where it interacts with transcription factors ABF4 and ABI5, reducing their binding activity to cis-regulatory sequences in ABA signaling downstream genes to attenuate ABA signaling [44]. Moreover, upon treatment with the rapid alkalinization factor 1 (RALF1) peptide, the receptor-like kinase FER can phosphorylate FREE1 at residues Ser265, Ser266, Ser268, Tyr269, Ser277, and Ser279, also promoting its shuttling to the nucleus to suppress ABA signaling [45]. In addition, plant-specific casein kinase I members MLKs 1–4 were elucidated to phosphorylate FREE1 at the same residues as SnRK2.2 and SnRK2.3 to promote its nuclear accumulation, thereby attenuating ABA signaling [46]. FREE1 also modulates vacuole fragmentation in root meristem cells to enhance plant salt tolerance. SOS2 phosphorylates FREE1 at residues Ser92, Ser216, Ser218, and Thr329, leading to its degradation and affecting MVB maturation, thereby reducing MVB–vacuole fusion in response to salt stress. As a result, MVB–vacuole fusion is inhibited, leading to vacuole fragmentation in response to salt stress. This vacuole fragmentation increases the surface-to-volume ratio of the vacuolar membrane, which may contribute to a more effective Na^+^ compartmentalization and enhance plant salt tolerance [47,48].

Ubiquitination mediated by E3 ligases targets substrate protein FREE1 for proteasomal or vacuolar degradation, whereas phosphorylation modulates FREE1 protein stability or subcellular localization. Different phosphorylation sites appear to dictate the divergent functional roles of the FREE1. However, SnRKs and MLKs phosphorylate FREE1 at residues distinct from those targeted by FER, and all these phosphorylation events drive FREE1 translocation into the nucleus. Moreover, the co-ordination between ubiquitination and phosphorylation likely plays a pivotal role in regulating FREE1 function, thereby ensuring precise control of abiotic stress signaling dynamics. For instance, SOS2-mediated phosphorylation of FREE1 triggers its degradation via the ubiquitin–proteasome pathway. Whether the reported E3 ubiquitin ligases of FREE1, the SINAT proteins, are involved in this process remains unclear. Additionally, the degradation pathway of nuclear FREE1 remains uncharacterized, whereas cytosolic FREE1 degradation induced by SOS2-mediated phosphorylation occurs via the 26S proteasome. Finally, while VPS23A is known to undergo degradation through both the 26S proteasome and vacuolar pathways, whether VPS23A is subject to phosphorylation requires further investigation.

Beyond regulating ABA signaling, FREE1 is involved in the salt stress response pathway. The phosphorylation site of FREE1 targeted by FER is also found to likely function in plant salt stress tolerance, indicating that the RALF1-FER-FREE1 module may co-ordinate plant growth and salt stress responses [45]. Additionally, FREE1 can form liquid-like condensates independently of ESCRT functions to drive ILV formation. FREE1 condensates alone are sufficient for plant survival under standard conditions, but osmotic tolerance requires a functional link between condensate-dependent and ESCRT-machinery-dependent MVB pathways [17]. However, the interplay between condensates and the ESCRT machinery in responding to osmotic stress remains unclear, representing an intriguing direction for future research.

Besides that, FREE1 also functions in other stress responses and physiological processes, including alkaline stress adaptation, iron deficiency tolerance, and amino acid sensing. As a key ESCRT subunit, it mediates vacuolar trafficking of PIN7, an essential process for root responses to alkaline stress. Its silencing disrupts PIN7’s vesicular transport and reduces sensitivity to alkaline stress, highlighting its indispensable role in this pathway [49]. In the context of iron homeostasis, FREE1 regulates IRT1-dependent metal transport and balance by controlling IRT1’s endosomal recycling to the plasma membrane and polar delivery to the outer plasma membrane domain, ensuring proper iron uptake and preventing metal toxicity; notably, monoubiquitination of IRT1 is critical for its internalization [50]. Under iron-deficient conditions, induced SINATs ubiquitinate FREE1 to promote its degradation, relieving FREE1-mediated repression [42], while the E2 conjugating enzyme UBC18 also co-operates with the E3 ligase SINAT1 to ubiquitinate and destabilize FREE1 [51]. Beyond these roles, FREE1 acts as an intracellular amino acid sensor that triggers target of rapamycin complex 1 (TORC1) activation, a function independent of its previously reported ESCRT-dependent activities [52].

## Other ESCRT components in plant abiotic stress

In addition to VPS23A and FREE1, many other ESCRT components and associated proteins are involved in plant abiotic stress response. Discrete TOLs proteins, which are recently reported as functional ESCRT-0 complex substitutes in plants, regulate the trafficking for degradation of core components of the ABA signaling and transport machinery. TOL2, TOL3, TOL5, and TOL6 modulate ABA signaling via mediating degradation of ubiquitinated ABA receptors and transporters, including PYR1, PYL4, and ABCG25. The *tol2/3/5/6* quadruple mutant is significantly more drought-tolerant and has a higher ABA sensitivity than the wildtype, with no obvious growth or development phenotype under standard conditions [12]. ALIX regulates ABA signaling by interacting with ABA receptor. Similar to VPS23A and FREE1, ALIX also affects the stability of ABA receptors by directly binding to ABA receptors PYL4 in late endosomes, promoting their degradation. *alix-1* mutation impairs the interaction with PYL4 and the ESCRT-I component VPS23A, hampering the internalization of PYL4 into ILVs in MVBs [27]. Besides affecting ABA receptors, ALIX affects endosomal localization of vacuolar sorting receptors (VSRs). ALIX functions as a unique retromer core subcomplex regulator by orchestrating receptor-mediated vacuolar sorting of soluble proteins. Depletion of ALIX perturbs membrane recruitment of Vps26 and Vps29 and alters the endosomal localization of VSRs [33]. In addition, Ca^2+^-dependent lipid binding protein 1 (CaLB1) interacts with AUTOPHAGY8 (ATG8) and PI(3)P, a phospholipid enriched in autophagosomal membranes. ALIX interacts via its C-terminal disordered region (IDR) with the CaLB1 C2 domain and binds to ATG8 proteins and PI(3)P, facilitating molecular aggregate formation. This mechanism recruits ESCRT-III complex to autophagosomes, modulates its activity, and co-ordinates ESCRT-mediated autophagosome maturation through either FREE1-dependent or FREE1-independent pathways, thereby enhancing plant salt tolerance. Furthermore, CaLB1 enhances phase separation of ALIX, facilitating the recruitment of ESCRT-III to the site of phagophore closure, thereby ensuring efficient maturation of autophagosomes [28]. The ESCRT-III component FYVE4 enhances SOS1 phosphorylation by promoting SOS1 and SOS2 interactions during salt stress. This extends beyond its established role in membrane trafficking regulation [20].

The increased abundance of LIP5 may enhance the activity of VPS4/SKD1, thereby helping to improve the tolerance of plants under adverse conditions. LIP5 mediates MVB pathways. In contrast to mutants of VPS4/SKD1, whose mutations are lethal, *LIP5* knockout mutants exhibit normal growth and development. This indicates that the activity of SKD1 ATPase is sufficient to support MVB biosynthesis required for Arabidopsis growth and development even without LIP5-mediated activation. However, the *lip5* mutants are compromised in stress-induced MVB formation, highly susceptible to microbial pathogens and sensitive to abiotic stresses such as salt and heat [21]. Thus, LIP5 acts as a key regulator of stress-induced MVB biosynthesis in plants in response to both biotic and abiotic stresses [22]. The LIP5 protein is unstable under normal growth conditions but becomes stabilized through phosphorylation by stress-responsive MAPK3 and MAPK6, thereby promoting stress-induced MVB biosynthesis. Additionally, it regulates the transport of defense-related molecules via MVBs or exosome-like paramural vesicles. Furthermore, another finding highlights LIP5’s role in salt tolerance by revealing its genetic and physical interaction with Increased Salt Tolerance 1-LIKE1 (ISTL1), a predicted homolog of yeast IST1 [53]. Hypertonic stresses enzymatically induce significant upregulation of MPK3 mRNA levels [54,55]. These results support the role of LIP5-mediated MVBs in actively responding to osmotic stress.

## Conclusions and future perspectives

The ESCRT machinery, as a conserved and plant-adapted endomembrane regulatory system, plays multifaceted roles in plant abiotic stress responses, integrating diverse signaling pathways and cellular processes to enhance stress adaptation. Key findings highlight that core components like VPS23A and the plant-specific FREE1 act as central hubs in this network. Both VPS23A and FREE1 play key roles in modulating ABA signaling and ABA-related drought, osmotic, or salt stresses through endosomal trafficking pathway-mediated vacuole protein. Meanwhile, FREE1 exhibits remarkable functional versatility: it also participates in alkaline stress adaptation, iron homeostasis, and amino acid sensing. Other ESCRT-associated proteins, such as ALIX and LIP5, further expand this regulatory network by influencing ABA signaling, autophagy maturation, and stress-induced MVB biogenesis. Collectively, these insights underscore the evolutionary conservation of ESCRTs alongside plant-specific innovations, enabling tailored responses to various environmental stresses.

Despite these advances, several critical questions remain to be addressed. First, most studies focus on ABA, drought, and salt stress, while investigations into heat, cold, alkalinity, and other stresses are extremely limited. Screening ESCRT components responsive to these stresses in model plants, such as *Arabidopsis* and rice, represents a promising approach for this field. The studies of ESCRTs in other abiotic stresses will help in addressing complex abiotic stresses. Second, while existing studies have highlighted the importance of specific ESCRT and related proteins, such as VPS23A, FREE1, LIP5, TOLs, and ALIX, in plant responses to abiotic stresses, much remains to be explored regarding the functions of other ESCRT components and also the plant-specific ESCRT components. Third, the full spectrum of post-translational modifications, including ubiquitination and phosphorylation, in regulating ESCRT components via specific modifying enzymes under distinct stresses demands systematic investigation. Finally, most of the studies were limited in the model plant Arabidopsis, while their function in crops is extremely limited. Translating these insights into practical applications, such as engineering ESCRT-mediated pathways to enhance crop stress tolerance, requires validating target genes in diverse crop species and optimizing their modulation to balance stress resilience with overall growth performance. For example, the *tol2/3/5/6* quadruple mutant plant line is significantly more drought-tolerant than control plant lines, with no obvious growth or development phenotype under standard conditions, making the *TOL* genes ideal candidates for engineering to improved plant performance [12].

Future research directions should appropriately use advanced technology. Structural biology studies focusing on key ESCRT components such as VPS23A and FREE1 proteins can offer valuable insights. Integrating techniques like primer editing and base editing for genome modifications including promoter, upstream ORF, and coding region at various scales, such as point mutations, short and long fragment insertion or deletion could present a promising strategy [56–58]. Precision editing may effectively prevent and mitigate growth and developmental defects caused by knockouts or overexpression. For example, *FREE1* knockout plants show seedling lethality; thus, precise modification of *FREE1* is a prerequisite for its application in crop improvement. In addition, if direct manipulation of key components proves challenging, their regulators or associated factors can be targeted for practical applications. These technologies have reached a relatively mature stage in rice and wheat, with expectations that their applications will soon extend to other crops. Addressing these questions will deepen our understanding of ESCRT-mediated stress adaptation and provide valuable targets for breeding abiotic stress-tolerant crops.

**Short Summary**

- The endosomal sorting complex required for transport (ESCRT) machinery sorts membrane proteins for degradation/recycling, affecting plant development and stress adaptation.
- ESCRT-mediated vacuolar degradation and ubiquitin–proteasome system regulate plant abiotic stress responses, especially via abscisic acid signaling.
- FREE1 mediates endosomal membrane bending and scission independent of ESCRT machinery.
- Further study of ESCRTs in abiotic stress adaptation may aid in breeding crops balancing yield and stress tolerance.

## Open Access

This article has been published open access under our Subscribe to Open programme, made possible through the support of our subscribing institutions, learn more here: https://portlandpress.com/pages/open_access_options_and_prices#conditional

## Abbreviations

ALIX: apoptosis-linked gene-2 interacting protein X)
BRAF: BRo1-domain protein as FREE1 suppressor
DUBs: deubiquitinases
ERAD: endoplasmic reticulum associated protein degradation
ESCRT: endosomal sorting complex required for transport
ILVs: intraluminal vesicles
LIP5: Vps twenty-associated 1/lyst-interacting protein5
MVBs: multivesicular bodies
PI(3)P: phosphatidylinositol-3- phosphate
PROS: positive regulator of SKD1
PYL: PYR-like
PYR: pyrabactin resistance
SPR: surface plasmon resonance
TOLs, TOM1-like proteins: SH3P2, Src homology-3 (SH3) domain-containing protein 2
TOR: target of rapamycin
UPS: ubiquitin–proteasome system
VPS4/SKD1: vacuolar protein sorting 4/suppressor of K^+^ transport growth defect 1
